# A comparative analysis of the fluorescence properties of the wild-type and active site mutants of the hepatitis C virus autoprotease NS2-3

**DOI:** 10.1016/j.bbapap.2009.10.006

**Published:** 2010-01

**Authors:** Toshana L. Foster, Philip R. Tedbury, Arwen R. Pearson, Mark Harris

**Affiliations:** Institute of Molecular and Cellular Biology, Faculty of Biological Sciences and Astbury Centre for Structural Molecular Biology, University of Leeds, Leeds LS2 9JT, UK

**Keywords:** HCV, Hepatitis C virus, NS, non-structural, JFH-1, Japanese fulminant hepatitis C virus genotype 2a isolate, WT, wild-type, GdnHCl, guanidine hydrochloride, DTT, dithiothreitol, CHAPS, 3-[(3-cholamidopropyl)dimethylammonio]-1-propanesulfonate, CD, circular dichroism, Hepatitis C virus, NS2-3 autoprotease, Mutagenesis, Refolding, Tryptophan fluorescence, Acrylamide quenching

## Abstract

Hepatitis C virus encodes an autoprotease, NS2-3, which is required for processing of the viral polyprotein between the non-structural NS2 and NS3 proteins. This protease activity is vital for the replication and assembly of the virus and therefore represents a target for the development of anti-viral drugs. The mechanism of this auto-processing reaction is not yet clear but the protease activity has been shown to map to the C-terminal region of NS2 and the N-terminal serine protease region of NS3. The NS2-3 precursor can be expressed in *Escherichia coli* as inclusion bodies, purified as denatured protein and refolded, in the presence of detergents and the divalent metal ion zinc, into an active form capable of auto-cleavage. Here, intrinsic tryptophan fluorescence has been used to assess refolding in the wild-type protein and specific active site mutants. We also investigate the effects on protein folding of alterations to the reaction conditions that have been shown to prevent auto-cleavage. Our data demonstrate that these active site mutations do not solely affect the cleavage activity of the HCV NS2-3 protease but significantly affect the integrity of the global protein fold.

## 1 Introduction

Hepatitis C virus (HCV) currently infects an estimated 170 million individuals worldwide; in approximately 80% of these cases, the virus establishes a chronic infection that leads to liver cirrhosis and hepatocellular carcinoma [1]. HCV infection is a growing health problem with an estimated three to four million individuals becoming newly infected each year. HCV infection is the leading indicator for liver transplantation in the developed world therefore research into identifying targets for therapeutic intervention is vital and has been stimulated by the limited ineffective therapy options and the lack of vaccines. [1,2].

**Fig. 1:**
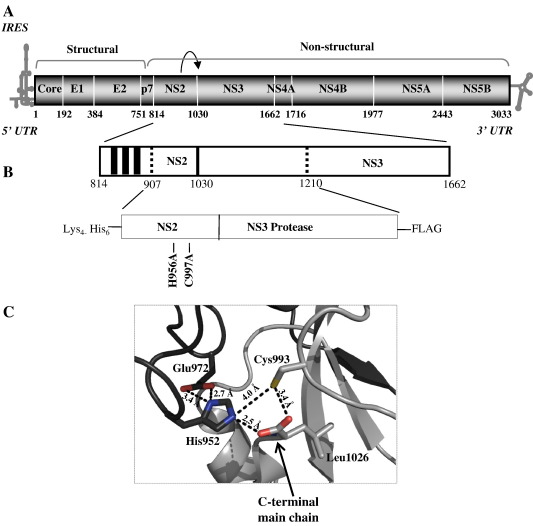
(A) Schematic representation of the HCV polyprotein. The HCV genome is translated into the structural proteins located at the N-terminus and the non-structural proteins at the C-terminus. Distances along the polyprotein are shown in amino acid residues. UTR, untranslated region; IRES, internal ribosomal entry site; E, envelope glycoprotein; NS, non-structural protein. Processing of the polyprotein precursor in the structural region and at the N-terminus of NS2 is mediated by host signal peptidase. The virus-encoded protease NS3 is responsible for further cleavages in the non-structural region of the polyprotein. The NS2–NS3 junction is cleaved by the NS2/3 autoprotease (denoted by the black cyclic arrow). (B) Schematic representation of the NS2-3 cleavage site. The region from residues 907–1210 maps to the minimal region required for autoproteolysis; numbering is based on the HCV genotype 2a JFH-1 isolate. This fragment was expressed with N-terminal hexahistidine and C-terminal FLAG-tags. The active site residues at which single point mutations were made in NS2 are labelled. (C) The active site of the domain swapped NS2 post-cleavage dimer of genotype 1a NS2-3. The figure was generated using Pymol version 0.99 and the coordinates of PDB code 2hd0 [20]. Residues His952, Glu972 and Cys993 are shown in stick representation. Carbon and backbone ribbon are coloured in black for monomer A and grey for monomer B. The C-terminal main chain of LLeu1026 of monomer B is coordinated to H952 and C993 in the post-cleavage domain swapped NS2-3 dimer.

HCV is an enveloped virus with a positive sense RNA genome of 9.6 kb consisting of a 5′-untranslated region (UTR) which includes an internal ribosome entry site (IRES), a single open reading frame (ORF) that encodes the structural and non-structural viral proteins and a 3′-UTR [2-5]. Cap-independent translation of the viral genome yields a large polyprotein of approximately 3000 amino acid residues [5,6]. Co-translational and post-translational processing of the polyprotein by host signal peptidases and two viral proteases at the endoplasmic reticulum (ER) results in 10 mature virus proteins; these cleavage products are ordered from the amino to the carboxy terminus as follows: core (C), envelope protein 1 (E1), E2, p7, non-structural protein 2 (NS2), NS3, NS4A, NS4B, NS5A and NS5B (Fig. 1A) [2,5-7]. Host signal peptidase cleaves the precursor polyprotein at the Core/E1, E1/E2, E2/p7 and p7/NS2 junctions [8], while the HCV-encoded NS3-NS4A serine protease complex cleaves the junctions of non-structural proteins NS3-5B [9]. Auto-processing by the virally encoded NS2-3 protease is responsible for cleavage at the NS2/NS3 junction; this activity has been demonstrated to be essential for replication of full-length virus and subgenomic replicons that span the NS2-NS5B region (Fig. 1A) [10,11]. Auto-cleavage by NS2-3 is a key step in the viral life cycle and is therefore a potential drug target, but little is known regarding its pre-cleavage protein structure, molecular mechanism and the regulation of this process.

Auto-cleavage of the NS2-3 precursor at the NS2/3 junction results in the release of the NS2 and NS3 proteins. NS2 is a 217 residue protein; the N-terminal 96 residues are highly hydrophobic and are proposed to form either three or four transmembrane helices that insert into the ER membrane [12]*.* The C-terminal region of NS2 resides in the cytoplasm and, together with the N-terminal region of the serine protease NS3, forms the NS2-3 autoprotease. This region (amino acids 907-1210 in HCV genotype 2a), mapped through truncation analyses, is the minimal region required for activity; NS2-3 cleavage activity occurs independently of NS3 protease activity (Fig. 1B) [13].

Biochemical and mutational characterisation has identified several determinants of NS2-3 cleavage [10-13]. In vitro processing at the NS2-3 site requires the presence of microsomal membranes or detergents suggesting that a hydrophobic environment is essential for the correct folding of the protease and for the subsequent cleavage. An in vitro assay based on the dilution of reduced and denatured NS2-3 into a cleavage buffer which contains high salt and glycerol as well as zwitterionic or non-ionic detergents can be used to assess cleavage of the NS2-3 junction by western blotting. The resulting in vitro cleavage is temperature sensitive and cleavage is greatly retarded at temperatures above 30 °C and below 15 °C [14-18].

Mutations around the highly conserved cleavage site 1023-WRLL⁎APIT-1030 are well tolerated, except for mutations that specifically disrupt the conformation of the NS2/3 junction such as the substitution of leucine and alanine for proline which rigidifies the peptide [16]. The serine protease activity of the mature NS3 protein is not required for the NS2-3 cleavage reaction; however, mutation of the three cysteine residues (Cys1123, Cys1125, Cys1171), which in the NS3 structure along with a histidine residue (His1175) are involved in the tetrahedral coordination of a Zn^2+^ ion, completely abolishes NS2-3 activity [10,13]. In addition, the activity of the enzyme can be inhibited by the presence of EDTA and stimulated by the addition of excess zinc further suggesting that the structural role of the coordinated Zn^2+^ ion is functionally important for NS2-3 activity [13]. Site-directed mutagenesis studies have identified two distinct residues within NS2 alone which are important for the activation of the protease, numbered in genotype 1b as His952 and Cys993 [13,15,17,18], and recently, an intrinsic basal enzymatic activity of NS2, followed by only two amino acids of NS3, has been identified, confirming that NS2 alone possesses proteolytic activity with NS3 acting as a stimulatory cofactor [19].

The three-dimensional structure of the N-terminal truncated NS2 portion (residues 904–1026 of genotype 1a) of the NS2-3 enzyme has been elucidated (Fig. 1C) [20]. It represents the post-cleavage state of the enzyme, as the C-terminal Leu1026 is coordinated within the proposed active site. It shows that NS2 forms a homodimer with two composite active sites: residues His952 and Glu972 from one monomer and Cys993 from the other [17-20]. From this crystal structure, it was proposed that NS2 is a cysteine protease based on the similarities in the spatial arrangement of these active site residues to known cysteine proteases such as papain and poliovirus 3C protease [20]. Residues His952, Glu972 and Cys993 within the putative active site are conserved between genotypes and mutations within this site result in inhibition of in vitro cleavage of the NS2-3 precursor (Fig. 1C) [13,14-16]. However, in the absence of the zinc-coordinating NS3 protease domain, this structure solely represents the post-cleavage state of the enzyme and raises questions, due to its dimeric nature, about the molecular level at which cleavage occurs during translation and polyprotein processing.

Key questions concerning NS2-3 biology relate to the understanding of the autoprotease mechanism and the structural changes that occur to accommodate cleavage. Current knowledge of the structural features of the NS2-3 precursor is very poor. This is mainly due to the strict conditions required for NS2-3 cleavage in vitro; i.e., high salt, glycerol and detergent content which often hampers X-ray crystallography and NMR spectroscopy techniques. The identification of active site residues, His952 and Cys993, which on mutation abrogate cleavage has aided some understanding of the activity of NS2-3 and are therefore targets for structural information on the pre-cleavage state of the enzyme. The effects these mutations have on the conformational rearrangement of the catalytically active precursor, i.e., the correct fold of the precursor state have not been addressed.

Here, we analysed the NS2-3 protease of genotype 2a isolate JFH-1 with selected NS2-3 point mutants, biochemically and by tryptophan fluorescence spectroscopy. This technique was used to independently assess the cleavage and folding of the wild-type and mutant autoprotease proteins to investigate conformational changes that occur upon refolding and to investigate global differences between the folds of the autoproteases showing that loss of enzymatic function is often associated with loss of protein folding.

## 2 Materials and methods

### 2.1 Expression and purification of recombinant NS2-3 autoprotease

The preparation of the JFH-1 NS2-3 wild-type and protease mutants His956-Ala (H956A) and Cys997-Ala (C997A), equivalent to H952A and C993A in the genotype 1b J4 isolate, and their expression via the modified pET23a vector in *Escherichia coli* BL21 (DE3) *pLysS*, has been described previously [13,18]. Briefly, *E. coli* BL21 (DE3) pLysS cells, harbouring the pET23a-NS2-3 constructs, were grown in LB medium supplemented with 1% glucose and 50 μg/μl ampicillin. Overexpression was induced at OD_600_ = 1 by the addition of 1 mM isopropyl-beta-d-thiogalactopyranoside (IPTG) and cells were grown for a further 3 h at 37 °C. The overexpressed proteins were recovered in inclusion bodies and solubilised in extraction buffer (6 M GdnHCl, 0.5 M NaCl, 50 mM Tris–HCl, pH 8.0) [13,18]. The inclusion bodies were clarified by centrifugation at 40 000×*g* for 1 h and the supernatant passed through a 0.22-μm filter. The filtrate was diluted 1:1 with extraction buffer supplemented with 40 mM imidazole and added to 1 ml of pre-charged chelating sepharose resin per 30 ml sample (Chelating Sepharose™ Fast Flow—Amersham Biosciences; the resin was charged with Ni^2+^ prior to use, according to manufacturer's instructions). The sample was incubated for 1 h at 4 °C then the resin was pelleted by centrifugation for 2 min at 1500×*g* . The resin was washed by resuspension and mixing for 5 min at 4 °C, twice in 10 ml extraction buffer and three times in 10 ml wash buffer (6 M GdnHCl (Sigma), 0.5 M NaCl, 50 mM imidazole, 50 mM Tris–HCl, pH 8). The protein was then eluted by resuspension and mixing for 5 min at 4 °C in 5 ml elution buffer (6 M GdnHCl, 0.5 M NaCl, 500 mM imidazole, 50 mM Tris–HCl, pH 8).

### 2.2 NS2-3 protease auto-cleavage assay

Denatured NS2-3 was supplemented with 100 mM DTT before diluting 1:100 with cleavage buffer that supports refolding (30% glycerol, 0.5% CHAPS, 250 mM NaCl, 3 mM cysteine, 50 μM ZnCl_2_, 50 mM HEPES–NaOH, pH 7). The reaction was incubated overnight at room temperature and stopped by the addition of an equal volume of 2× Laemmli sample buffer (100 mM Tris–HCl pH 6.8, 20% glycerol, 4% SDS, 0.04% bromophenol blue, 10 mM DTT). The cleavage reaction was analysed by 15% SDS–PAGE and western blot using M2 anti-FLAG monoclonal antibody (Sigma), followed by an HRP-conjugated goat anti-mouse antibody (Sigma). Where appropriate, western blotting was performed with sheep polyclonal sera raised to NS3 followed by an HRP-conjugated donkey anti-sheep antibody (Sigma).

### 2.3 Glutaraldehyde cross-linking reactions

Glutaraldehyde cross-linking was performed by incubating refolded NS2-3 precursor (0.2 μM) with freshly prepared glutaraldehyde solution (Sigma, final concentrations, 0.001–0.002% [v/v] in refolding buffer) in a 50-μl reaction mixture at 25 °C for 5 min. The reaction was quenched by the addition of an equal volume of 2× Laemmli sample buffer before cross-linked products were analysed by SDS–PAGE and western blot using M2 anti-FLAG monoclonal antibody (Sigma), followed by an HRP-conjugated goat anti-mouse antibody (Sigma).

### 2.4 Tryptophan fluorescence spectroscopy

All tryptophan fluorescence emission spectra were recorded on a Fluorolog®-3 (HORIBA JOBIN YVON) fluorescence spectrometer. Slit widths of 3 nm were used on the emission monochromator and temperatures were set to 25 °C or 4 °C accordingly. The protein concentration used for measurements was 50 μM. Measurements were performed in a 10-mm path length cell by illuminating NS2-3 precursor samples with 295-nm wavelength light in order to selectively excite the tryptophan residues of the protein. The emission spectra were recorded between 290 and 500 nm.

### 2.5 Circular dichroism measurements

CD measurements were recorded on a Jasco J175 spectropolarimeter (Jasco International Co. Ltd) equipped with a Peltier thermostat. The concentration of the protein used was maintained at 6 μM in the denaturing buffer or refolding buffer for measurements in the far-UV region (200–260 nm). For the refolding conditions, HEPES buffer was replaced by 50 mM sodium phosphate pH 7.0 and 100 mM DTT was substituted by 10 mM beta-mercaptoethanol. Measurements were recorded in a 1 mm path length cell with 0.5-nm increments and 4-s integration times and averaged over 3 scans. Buffer subtraction was used for baseline correction and spectral units were converted to molar ellipticity per residue using the protein concentrations determined by amino acid analysis.

### 2.6 Acrylamide quenching studies

For quenching of intrinsic tryptophan fluorescence, aliquots of 5 M quencher stocks of the external quencher acrylamide (Sigma) prepared in either 6 M GdnHCl, 100 mM Tris–HCl, pH 8.0, or in NS2-3 cleavage buffer were added to the protein samples and fluorescence spectra were recorded after each addition. The final quencher concentration attained in each case was 0.5 M. Each quenching reaction was set up separately to account for dilution effects and reactions were performed in triplicate at 4 °C. The quenching data were analysed by the Stern–Volmer equation: *F*_0_/*F* = 1 + *K*_SV_[*Q*], where *F* and *F*_0_ are the relative unquenched and quenched intensities, *K*_SV_ is the Stern–Volmer constant and *Q* is the concentration of the quencher in solution. For a heterogeneous population of emitting fluorophores, the plot of *F*_0_/*F* versus [*Q*] is non-linear. To estimate the fraction of accessible fluorophore quenched with a Stern–Volmer constant *K*_SV__,_ modified Stern–Volmer plots were used, as according to Lehrer and Leavis [21]:

(*F*_0_/(*F*_0_ *−* *F*) = 1 / [*Q*]*f*_a_*K*_SV_ + 1 / *f*_a_), where *f*_a_ is the fractional accessible fluorescence determined from a plot of *F*_0_/(*F*_0_ *−* *F*) versus 1/[*Q*].

## 3 Results

### 3.1 Characterisation of JFH-1 NS2-3 precursor

**Fig. 2:**
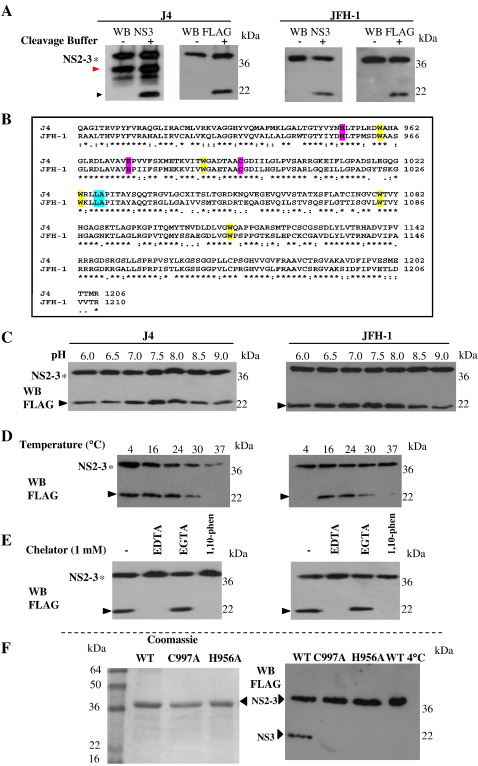
Comparative analyses of recombinant WT NS2-3 precursor proteins derived from J4 or JFH-1 isolates. (A) The cleavage activity of the J4 and JFH-1 precursors was probed by western blotting using anti-NS3 polyclonal sera and anti-FLAG monoclonal antibody. Genotype 2a JFH-1 NS2-3 lacks the truncation product of genotype 1b J4 NS2-3 detected by anti-NS3 antibody. The black triangle indicates the NS3 cleavage product while the red triangle indicates the truncation product observed in J4 NS2-3. (B) Sequence alignment from Clustal W2 multiple alignment of genotype 1b strain J4 NS2-3 (GenBank accession no. AF054250) and genotype 2a strain JFH-1 NS2-3 (GenBank accession no. AB047639). The tryptophan fluorophores are highlighted in yellow and the cleavage site in cyan and active site residues in magenta. (C) The pH of the cleavage buffer was varied by 0.5 pH units within the range from pH 6.0 to pH 9.0 to assess the dependence of cleavage activity on pH for the two proteins. (D) The temperature dependence of cleavage activity between J4 and JFH-1 NS2-3 was compared over a 16-h period. Optimal activity at 24 °C for both proteins and inactivity of JFH-1 at 4 °C compared to J4 as observed. (E) The inhibition of auto-processing of NS2-3 by the chelation of metal ions was assessed by the addition of EDTA, EGTA and 1,10-phenanthroline (1,10-phen) to the cleavage reactions at 1 mM concentrations. (F) Wild-type NS2-3 precursor and the two active site mutants H956A and C997A were expressed and purified by nickel affinity chromatography and analysed by 15% SDS–PAGE followed by Coomassie staining. For auto-processing, purified wild-type NS2-3 precursor or the two mutants H956A and C997A were supplemented with 100 mM DTT, diluted 1:100 into cleavage buffer and incubated at 25 °C or 4 °C. For all data in panels C–F apart from the Coomassie stained gel in panel F, the products were separated by SDS–PAGE and probed by western blotting with anti-FLAG monoclonal antibody. Black triangles indicate the NS2-3 precursor and the NS3 protease domain cleavage product.

Previous biochemical studies of the NS2-3 protease have been performed with protein of the genotype 1b J4 isolate. One complication of the analysis of the activity and folding of J4 NS2-3 is the presence of a 30-kDa C-terminal truncation product of the full-length protease (Fig. 2A) [13], which is detectable with NS3 antisera but not detectable with the FLAG antisera, as the truncation lacks the FLAG-tag located at the C-terminus of the full-length protein. This abundant truncation product is non-functional but would contribute significantly to the data collected in analyses of the global folding of the sample by tryptophan fluorescence. This truncation product, however, is not detectable in preparations of JFH-1 NS2-3, making the NS2-3 protease from genotype 2a more suitable for the subsequent assessment of refolding of the functional, full-length precursor state.

Sequence alignment of the two proteins of differing genotype showed that there is high conservation of amino acid sequence in 67% of sequence positions (denoted by asterisks in Fig. 2B), with absolute conservation of the protease active site residues of NS2 and NS3, the cleavage site of the NS2-3 precursor and the positions of the tryptophan fluorophores. We have demonstrated through comparative analyses of the purified recombinant NS2-3 autoproteases from J4 and JFH-1 isolates that the proteins exhibit similar characteristics in terms of auto-cleavage requirements and efficiency. As shown in Fig. 2C, both J4 and JFH-1 autoproteases are active across a wide pH range with the highest cleavage activity observed between pH 7.0 and 8.0. Protease activity for both precursors is sensitive at and above 30 °C; however, J4 retains the ability to auto-cleave at 4 °C, while there is no activity of the JFH-1 protein at this temperature (Fig. 2D). Whether this inactivity is related to protein misfolding will be investigated later in the article. The activity of NS2-3 is sensitive to the chelation of the Zn^2+^ ion from NS3. The inhibition of cleavage of the two NS2-3 precursors by Zn^2+^ chelation was demonstrated using EDTA, 1,10-phenanthroline, both chelators of divalent metal ions and EGTA, a chelator with preference for calcium ions, all at 1 mM concentration. 1,10-phenanthroline does not, however, chelate calcium or magnesium ions. As demonstrated in Fig. 2E, unlike EDTA and 1,10-phenanthroline, EGTA does not inhibit auto-cleavage of either J4 or JFH-1 NS2-3, suggesting that Zn^2+^ coordination is important in the structural conformation and stability of the JFH-1 NS2-3 precursor [13]. As a result of these similarities with the genotype 1b J4 isolate, JFH-1 NS2-3 has been used for all spectroscopy data reported here.

### 3.2 Functional refolding of WT NS2-3 autoprotease

Wild-type (WT) NS2-3 precursor and H956A and C997A mutants were expressed in *E. coli* as N-terminal hexahistidine-tagged and C-terminal FLAG-tagged recombinant proteins. The proteins, of apparent molecular mass 35 kDa, were purified from insoluble inclusion bodies by solubilisation in GdnHCl and metal chelate affinity chromatography, as described previously (Fig. 2F) [13].

NS2-3 auto-cleavage was stimulated by dilution into a cleavage buffer which allowed refolding of the enzyme. Denatured NS2-3 precursor in 6 M GdnHCl, at 50 μM concentration, was first treated with 100 mM DTT to completely eliminate non-specific protein–protein interactions that contribute to aggregation. This denatured and reduced protein was then diluted 100-fold into cleavage buffer and incubated at 25 °C overnight. The efficiency of auto-cleavage of the refolded NS2-3 protein, at low protein concentrations, was monitored by western blotting (Fig. 2F, right-hand panel), with the appearance of the 19-kDa C-terminal NS3 fragment, detectable with antibodies to NS3 or the FLAG-tag, indicating successful auto-cleavage. The active site mutants H956A and C997A are shown to be inactive [10,11,15], as no cleavage product is observed upon comparison with the processing of the WT protein, which has an in vitro cleavage efficiency of approximately 30% (Fig. 2F).

**Fig. 3:**
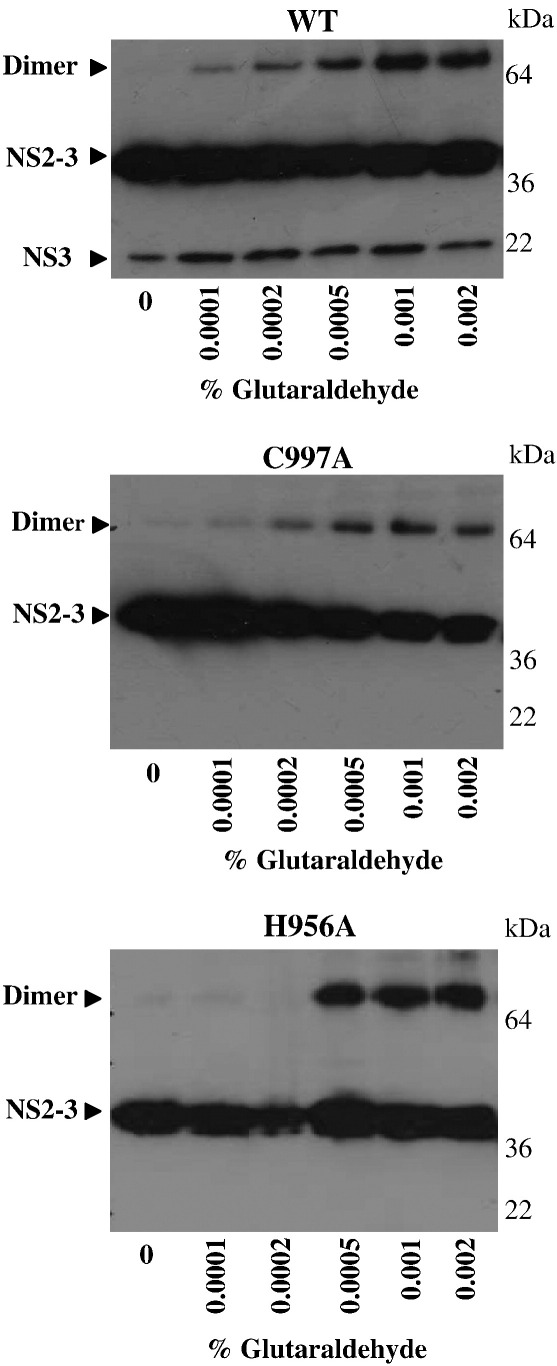
Glutaraldehyde cross-linking of refolded NS2-3 precursors. To analyse the dimerisation of NS2-3, the protein was treated with increasing concentrations of glutaraldehyde (final concentration 0.0001 to 0.002% [v/v]) and the NS2-3 protein complexes were analysed by SDS–PAGE and western blot.

The post-cleavage crystal structure of NS2 suggests that dimerisation is required for the activity of the autoprotease [20]. It is plausible that the inactivity of the two mutants was due to defects in the ability to dimerise. To test this, we investigated dimer formation by glutaraldehyde cross-linking (Fig. 3). As demonstrated by the appearance of increasing amounts of WT NS2-3 dimer, cross-linking increased in a dose-dependent manner with increasing concentrations of glutaraldehyde. It appears that the rate and the efficiency of dimerisation of WT NS2-3 and C997A are similar, but with great contrast to that observed with H956A which exhibited a lower efficiency for dimer formation. This initial observation highlights similarities between the inactive mutant C997A and WT NS2-3. Chemical cross-linking, however, is simply dependent on the likelihood that the reactive groups are close together; therefore, little information about the overall protein folds can be derived solely from these experiments.

**Fig. 4:**
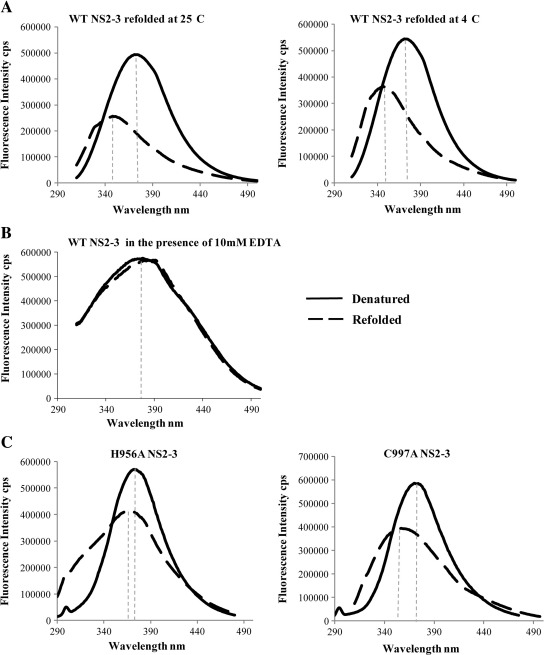
Fluorescence emission spectra of wild-type and mutant NS2-3 precursor. (A) Comparison of the fluorescence emission spectra of WT NS2-3 at 4 °C or 25 °C under denaturing (solid line), and refolded (dashed line), conditions. Low temperature inhibits activity but allows folding of the NS2-3 precursor. The vertical line indicates the tryptophan emission maximum of NS2-3. (B) Inhibition of activity by zinc chelation is directly linked to the lack of refolding in the presence of 10 mM EDTA. (C) Fluorescence emission spectra of the active site mutants H956A and C997A.

Direct comparison of any differences in the folding properties of the WT protease and the mutants requires analysis of WT NS2-3 in a pre-cleavage state. The auto-cleavage of JFH-1 NS2-3 protease, unlike J4 NS2-3, does not proceed at 4 °C (Fig. 2F); therefore, this made it possible to assess and compare the folding of WT and mutant NS2-3 pre-cleavage. To investigate whether refolding at low temperature had any differential effect on the conformation of the protease compared to that at 25 °C, we measured the fluorescence emission spectra of denatured and refolded WT NS2-3 precursor at 25 °C and at 4 °C after excitation at 295 nm. The emission spectra of denatured WT NS2-3 at the two temperatures show similar emission maxima (373 nm at 25 °C, 371 nm at 4 °C) and intensities demonstrating that the polarity of the microenvironment around the tryptophan residues of NS2-3 at the two temperatures is similar (Fig. 4A). Upon refolding overnight at 4 °C or 25 °C, wild-type NS2-3 exhibited both a decrease in the tryptophan fluorescence intensity and a blue shift of the maximal emission wavelength to 342 nm, indicating that the environment around the tryptophan residues had become more hydrophobic (Fig. 4A). The folding of the NS2-3 precursor therefore occurs at 4 °C; this temperature represents a suitable condition under which to study the conformation of the precursor state of the wild-type enzyme. Comparisons of the fluorescence properties of the WT protease with those of the mutants at 4 °C were therefore assessed and detailed in section 3.3.

WT NS2-3 has been reported to show no auto-cleavage in the absence of zinc [13]. The ability of the divalent metal ion chelator EDTA to inhibit auto-cleavage suggests that the presence of the Zn^2+^ ion within NS3 is required [13]. Addition of 10 mM EDTA to the cleavage buffer resulted in inhibition of both activity and folding of the enzyme; the fluorescence emission spectra after excitation at 295 nm showed that the NS2-3 precursor after overnight incubation remained in the unfolded state (Fig. 4B), consistent with the lack of cleavage observed by western blot (Fig. 2E). This finding provides direct evidence that zinc plays an important structural role in the correct folding of the NS2-3 precursor.

### 3.3 Active site mutations affect the global fold of the NS2-3 protease

The intrinsic tryptophan fluorescence of the wild-type enzyme was compared with those of the active site mutants H956A and C997A. On excitation of the denatured NS2-3 precursors at 295 nm, no significant differences in the peak emission wavelength around 377 nm were detected between the wild-type and the mutants demonstrating that the environment for each protein in the unfolded state is similar. It is expected that in all cases, the proteins will be fully extended in denaturant and therefore tryptophan residues will be exposed in this environment (Fig. 4A, C). However, the changes in the relative fluorescence intensities and shifts in the maximal emission wavelengths of the refolded mutants (370 nm for H956A and 358 nm for C997A) were less marked compared to the wild-type (Fig. 4A, C). The latter suggests that in contrast to wild-type NS2-3, upon incubation in refolding buffer, the tryptophan residues of the mutants remain exposed to a more polar environment; hence, the mutant precursor state is a more solvent-exposed conformation in comparison to the WT NS2-3 precursor. The highest emission maximum was observed for H956A signifying that the tryptophan residues in this particular protein conformation exist in a more polar environment relative to C997A and WT NS2-3.

**Fig. 5:**
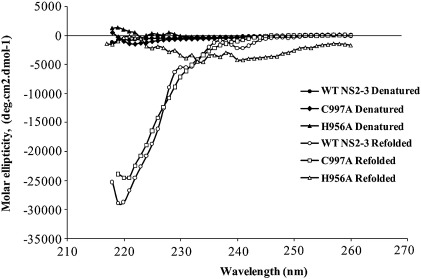
Far-UV CD spectra of WT NS2-3 and active site mutants C997A and H956A. The spectra were recorded at 4 °C under denaturing (6 M Gdn-Cl) and refolding buffer conditions.

The secondary structure characteristics of the NS2-3 precursor state were investigated by circular dichroism. Auto-cleavage under the refolding conditions used for the circular dichroism analysis, i.e., in the presence of sodium phosphate buffer and DTT, was assessed by western blot. Cleavage was observed as expected at room temperature but not at 4 °C (data not shown). The far-UV CD spectra of the denatured precursors show that no secondary structure characteristics can be observed in 6 M Gdn-Cl for any of the unfolded proteins (Fig. 5). Upon refolding, the NS2-3 precursors yielded very noisy CD spectra due to the high chloride ion, glycerol and detergent content of the refolding buffer especially below 215 nm. However, the shape and intensity of the spectra still allowed comparisons between the WT and mutant proteins. The spectra show that WT NS2-3 has some α-helical secondary structure characteristics with a minimum around 222 nm. The CD data also show that C997A has very similar secondary structure characteristics to the WT protein with the spectrum resembling that of the WT precursor state. H956A, however, appears to lack any secondary structure characteristics in the region of the spectrum that can be analysed, implying that H956A may be in an unfolded state and lack any α-helical similarities to the WT precursor (Fig. 5).

### 3.4 Quenching of the intrinsic fluorescence emission of NS2-3 precursors

The degree of accessibility of the tryptophan residues in the WT and mutant precursor states can be investigated by fluorophore quenching studies. Quenching experiments were carried out using the neutral quencher acrylamide, which is highly sensitive to the extent of tryptophan exposure to the solvent. Quenching of tryptophan fluorescence by acrylamide may result from accessibility of tryptophan residues on the surface of the protein or from the accessibility of residues through channels that lead to the interior of the protein.

**Fig. 6:**
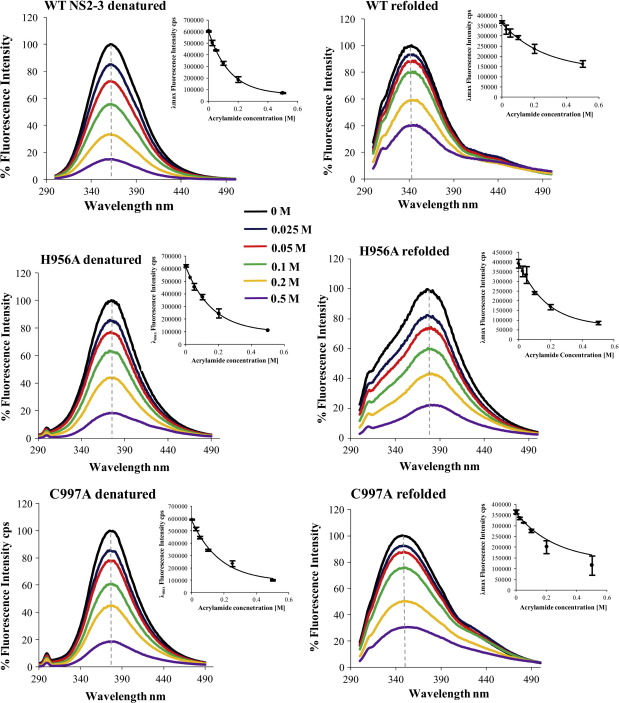
Fluorescence emission spectra of wild-type and mutant NS2-3 precursor in the absence and presence of acrylamide under denaturing conditions and refolded in cleavage buffer. The effect of increasing concentrations of acrylamide from a 5-M stock on the tryptophan emission of WT NS2-3 and mutants is represented as a percentage of the fluorescence at the maximal wavelength. Plots of percentage intensity against acrylamide quencher concentration are shown, where black = 0 M, blue = 0.025 M, red =  0.05 M, green =  1 M, orange =  0.2 M and purple =  0.5 M. Experiments were performed in triplicate and the standard errors among values at each acrylamide concentration are shown inset. Plots were fitted using the exponential equation *y* = *y*_0_ + *A*^e(*bx*)^ in OriginPro8 (OriginLab).

The fluorescence emission spectra of denatured and refolded wild-type NS2-3, H956A and C997A mutants were recorded in the absence and presence of increasing concentrations of acrylamide (Fig. 6); as expected, a progressive decrease in the fluorescence intensity was observed with increasing quencher concentration. The spectra of each NS2-3 precursor indicate that the extent of quenching is higher in the presence of GdnHCl, indicating that in the unfolded state the tryptophan residues are more accessible. The tryptophan residues in the refolded WT NS2-3 precursors are accessible to acrylamide, yet differences in the extent of quenching of C997A and H956A are observed, indicating that the fluorophores of WT NS2-3 are in a more hydrophobic environment. Notably, the extent of quenching achieved by acrylamide with refolded H956A is not significantly different to that of H956A in the denatured state. These data clearly indicate that the tryptophan residues are experiencing a similar environment when in denaturant and when under refolding conditions in this mutant.

**Fig. 7:**
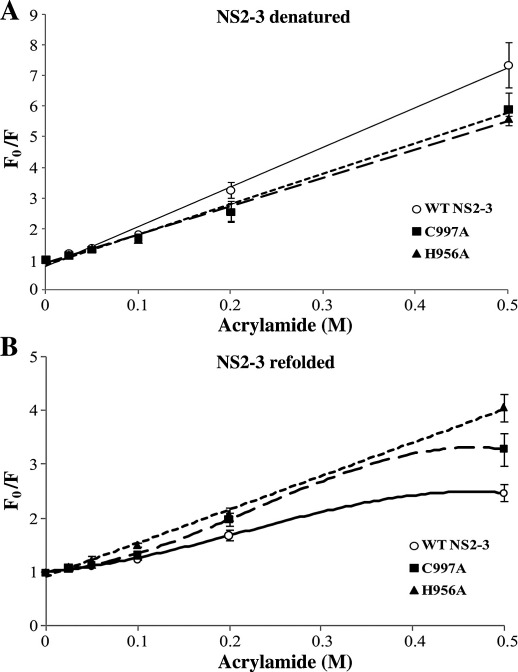
Stern–Volmer plots comparing the quenching of NS2-3 precursors WT (○), H956A (▴) and C997A (▪) in the presence of acrylamide in denatured (A) and refolded states (B). Plots were derived using the linear equation *y* = *a* + *bx* in OriginPro8 (OriginLab). The standard errors of the averages from three separate experiments are plotted. Under refolded conditions, non-linear Stern–Volmer relationships were observed for WT and C997A NS2-3; therefore, the accessibility of the tryptophan fluorophores to the quencher and the quenching constants under refolding conditions were derived from modified Stern–Volmer plots where *F*_0_/(*F*_0_ *−* *F*) versus 1/[*Q*] was plotted (data not shown).

The Stern–Volmer relationships for WT NS2-3 and mutants are plotted in Fig. 7 and quenching constants were determined for each NS2-3 protein in the denatured and refolded states (Table 1). In the denatured state, linear Stern–Volmer plots were observed for all proteins indicating that a homogenous population of fluorophores is being quenched. The NS2-3 precursors possess similar quenching patterns and share similar quenching constants demonstrating that comparable extended and flexible conformations are adopted by the NS2-3 precursors when in the presence of strong denaturant (Fig. 7A). As in the unfolded state, a linear Stern–Volmer relationship was observed for refolded H956A, in addition the highest quenching constant was observed for H956A (6.48 M^− 1^) (Fig. 7B), relative to WT and C997A, suggesting that the structure of this mutant is more open and dynamic allowing easy interaction of acrylamide with the tryptophan fluorophores. Upon refolding, biphasic or non-linear quenching patterns are observed for WT NS2-3 and C997A precursors, indicating that dynamic fluorescence quenching of multiple groups of tryptophan residues in different accessible environments is being observed (Fig. 7B). Modified Stern–Volmer plots were used to determine the fraction of accessible tryptophan fluorophores, *f*_a_, and the quenching constant *K*_SV_, for WT NS2-3and C997A by plotting *F*_0_/(*F*_0_ *−* *F*) versus 1/[*Q*] (data not shown). The plots derived were linear allowing derivation of the *f*_a_ and *K*_SV_ values. The data reported in Table 1 show that all of the tryptophan residues of H956A are accessible to the acrylamide quencher, *f*_a_ = 1.0. Contrastingly, a smaller fraction of the tryptophan residues of WT NS2-3 and C997A are observed to be accessible; *f*_a_ values =  0.57 and 0.75, respectively. These results are consistent with the CD data presented earlier showing that H956A does not have the secondary structure characteristics of the C997A and WT NS2-3 precursors.

## 4 Discussion

The NS2-3 precursor is an ideal target for the development of novel HCV anti-viral therapies. Structural information about the autoprotease precursor is required for understanding the protease mechanism and two mutants within NS2, His956A and Cys997A, have been identified as candidates for trapping NS2-3 in the precursor state. The crystal structure of the post-cleavage state shows His956 and Cys997 as part of a composite active site in an NS2 dimer, apparently reflecting a functional domain swapped NS2-3 dimer required for activity [20]. As a consequence, mutation of these residues to alanine (H956A and C997A) might be expected to have significant consequences not only for enzymatic function, but also for the formation of the catalytically active pre-cleavage protein conformation. This article describes the first biophysical characterisation of the precursor state of the NS2-3 protein and its active site mutants.

**Fig. 8:**
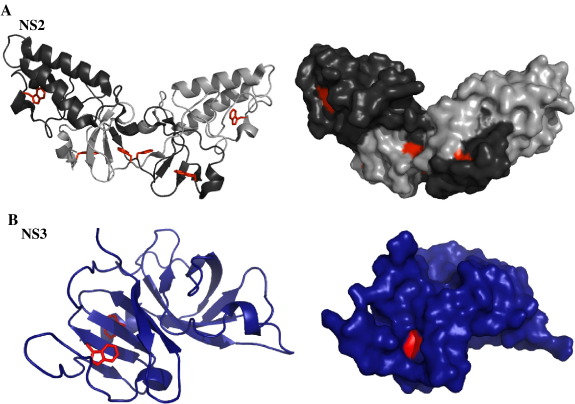
Location of the five tryptophan residues in the NS2 (A) and NS3 (B) subdomains of the genotype 1a NS2-3 precursor. The figure was generated using Pymol version 0.99 and the coordinates of PDB code 2hd0 (A) [20] and 1a1r (B) [24]. The tryptophan fluorophores are shown in red in stick representation and surface representation. Carbon and backbone ribbon are coloured in black for monomer A and grey for monomer B in the NS2 subdomain.

Using recombinantly expressed NS2-3 protein of genotype 2a, we characterised NS2-3 in its pre-cleavage state using low temperature and optimisation of the refolding conditions (Fig. 2). The microenvironment of protein residues is conditional on a variety of physical and chemical characteristics due to specific interactions, neighbouring residues, charged groups and polarizability, influencing the overall fluorescence of the protein. The restricted conditions required for NS2-3 refolding, i.e., high detergent, glycerol and salt concentrations, are not amenable to typical conformational and folding analysis by, for example, circular dichroism, Fourier transform infrared spectroscopy (FTIR) or dynamic light scattering techniques. Tryptophan fluorophores are, however, particularly sensitive to their environment; hence, these residues act as useful structural probes [22,23]. A total of five tryptophan residues are located in the NS2-3 precursor, three in the NS2 cytoplasmic domain and two in the NS3 protease domain; as shown in Fig. 8, it appears that in the folded post-cleavage structures of NS2 and NS3 [20,24], the tryptophan residues are in both buried and solvent-exposed regions. Here we have assessed the pre-cleavage conformation during refolding of the NS2-3 precursors under various conditions where the protein can undergo auto-cleavage, those where auto-cleavage is inhibited and the folding of the inactive mutants H956A and C997A. Importantly, performing the refolding reaction of WT NS2-3 at 4 °C prevented auto-cleavage but allowed conservation of the overall precursor fold, in comparison to the chelation of zinc which abrogated both cleavage and folding of the enzyme. The conformational changes that affect refolding in the case of C997A can also be distinguished from those observed for WT NS2-3 in the presence of the zinc chelator EDTA and from those of the mutants in which the cleavage site residues Leu1030 and Ala1031 have been changed to proline (LAPP) [11,13]—these exhibit a tryptophan fluorescence profile similar to that of H956A (data not shown).

The results presented here suggest that the inactive mutants have considerable differences in their tertiary structure around the tryptophan residues, possibly in both the NS2 and NS3 subdomains, in addition to the loss of auto-catalytic activity observed. The two active site mutants, however, do not appear to be experiencing the same global conformational changes in their structure during refolding as the fluorescence shifts observed for C997A are closer to those of the wild-type enzyme than to H956A. The CD measurements conducted were affected by the refolding buffer conditions required for NS2-3 refolding and activity. Despite this, it was observed that the secondary structure content of C997A is comparable to that of the WT NS2-3 precursor protein. H956A did not appear to possess any secondary structure characteristics under these conditions (Fig. 5). The increase in the *K*_SV_ value for the mutant precursor proteins from the wild-type value and the increase in accessibility of the quencher acrylamide to the core of the proteins indicated that the precursor state formed by the mutant NS2-3 proteins was in the incorrect conformation in which cleavage was unable to occur.

In cysteine proteases, the catalytic triad, in NS2-3 proposed to be C997-H956-E976 [20], forms a stable charge relay system that serves to increase the nucleophilic properties of the active cysteine and to stabilise the transition state [25]. As the active site mutations C997A and H956A abrogate activity, as assessed on the basis of in vitro function using gel based assays, this has been viewed as confirmation of their active site roles [15,17]. However, from the findings reported, it is clear that these two amino acids play an important role in maintaining the integrity of the active site conformation and this importance extends to the global fold of NS2-3 precursor. The mutant H956A, in particular, appears to cause severe disruption to protein folding in addition to the loss of enzymatic activity. From the post-cleavage structure, H956 makes many hydrogen bond contacts compared to C997, the loss of which would explain the loss of activity and fold on mutation. C997A appears to have a more native-like fold but the quenching experiments show that it is less tightly folded than the wild-type protease, making it a folding mutant. These data would also suggest that future studies aimed at elucidating structural information about the NS2-3 precursor would benefit from using C997A or low temperature to inhibit auto-cleavage, rather than H956A or the sequestration of zinc.

This study serves to illustrate a wider point that the loss of activity exhibited in mutants of putative active site residues may not solely result from a loss of catalytic activity but can also be related to gross changes in the global protein fold. Although C997A and H956A appear to be loss of function mutants, our detailed experiments reported here show that these mutants alone are insufficient for studying the activity and mechanism of the NS2-3 protease. Additional mutants that inhibit cleavage but maintain tertiary structure will need to be probed for further molecular level investigations into HCV NS2-3 biology, a conclusion that likely holds true for many proteins.

## Figures and Tables

**Table 1:** Accessibility of tryptophan residues of WT NS2-3 and active site mutants H956A and C997A to the quencher acrylamide.

NS2-3 precursor	Refolded		Denatured
	*K*_SV_	*f*_a_	*K*_SV_
Wild-type	2.76 ± 0.52	0.57 ± 0.3	12.9 ± 0.43
C997A	3.56 ± 0.48	0.75 ± 0.2	9.92 ± 0.41
H956A	6.48 ± 0.4	1.0 ± 0.3	9.22 ± 0.33

Both the fluorescence accessible to the quencher *f*_a_, and quenching constants *K*_SV_, are detailed for WT NS2-3 and active site mutants. Values are averages from three independent experiments and standard error values have been calculated.
